# Hypothesized pathways for the association of vitamin D status and insulin sensitivity with resting energy expenditure: a cross sectional mediation analysis in Australian adults of European ancestry

**DOI:** 10.1038/s41430-022-01123-4

**Published:** 2022-04-01

**Authors:** Mario J. Soares, Emily K. Calton, Kaveri Pathak, Yun Zhao

**Affiliations:** School of Population Health, Faculty of Health Sciences, Curtin University, Bentley Campus, Perth, 6102 Western Australia Australia

**Keywords:** Physiology, Endocrinology

## Abstract

**Background:**

The role of vitamin D in human energy expenditure requires confirmation. We explored whether insulin sensitivity (IS)/insulin resistance (IR) mediated the association of vitamin D status (25OHD) on resting energy expenditure (REE).

**Methods:**

REE, body composition (by DEXA) and clinical biochemistry of 155 Australian men and women were collated. A hypothesized mediation pathway through IS/IR on the direct association between 25OHD and REE was modeled, using three surrogate indices of IS/IR: McAuley’s insulin sensitivity index (McA), Quantitative insulin sensitivity check index (QUICKI) and triglyceride to glucose ratio (TYG). The modeling was performed on PROCESS SPSS Macro (version 4.0) based on 5000 bootstrapped samples, with and without the adjustment for covariates.

**Results:**

Unadjusted models indicated a sizeable negative mediation by all IS/IR indices but no significant direct effect of 25OHD on REE. On adjustment for covariates, a negative indirect mediation effect of McA [β coefficient (SE) −2.1(0.821); bootstrapped 95% CI:−3.934, −0.703; *p* < 0.05] and a similar negative mediation of TYG [−1.935 (0.780); bootstrapped 95% CI: (−3.679, −0.622; *p* < 0.05] was observed. These models also showed a positive direct effect of 25OHD on REE. In contrast, QUICKI made a smaller contribution to the total effect though in the same direction as the other two measures [−0.783 (0.534); bootstrapped 95% CI: (−1.939, 0.134; *P* > 0.05].

**Conclusions:**

A sizeable, partial, negative mediation of IS/IR on the direct relationship between 25OHD and REE, dampened the total effect of vitamin D on REE. Validation of the proposed causal framework would clarify vitamin D’s role in human energy metabolism.

## Introduction

Resting energy expenditure (REE) is the least amount of energy expended in the rested but awake state. It is the largest component of total energy expenditure [1], and hence forms the basis of estimating human energy requirements. After accounting for variables such as age, gender, detailed body composition and hormones, there remains a sizeable and unexplained variance (~15%) to REE [1]. Identifying and understanding additional factors that contribute to REE is important as they would fine-tune our understanding of energy balance, and potentially provide novel targets in the management of obesity.

The prevalence of vitamin D insufficiency in Australia is high [2], as it is in many other populations of the world [3]. There is growing evidence that vitamin D has many extra-skeletal roles [3–5], and these include an involvement in energy balance [6–10], insulin sensitivity (IS) [11–14], and the modulation of inflammation [15]. Achieving and maintaining a good vitamin D status, may then decrease the prevalence of obesity per se and many of its chronic sequlae. We had made the novel observations that vitamin D status (assessed by circulating 25OHD), as well as IS (as judged by McAuley’s insulin sensitivity index (McA)), were independent predictors of REE in a regression model [16]. While 25OHD was positively related to REE, McA was negatively associated with REE. Vitamin D receptors (VDR) are found on pancreatic islet β cells and act to increase insulin secretion [17], and several studies indicate that vitamin D supplementation reduced insulin resistance (IR) [11–14]. There is hence biological plausibility that vitamin D status may directly influence REE, or act indirectly via IS/IR, and thereby effect energy metabolism. Determining the overall effect and the relative contributions of 25OHD and IS on REE, needed clarification and was the focus of the analysis.

**Fig. 1:**
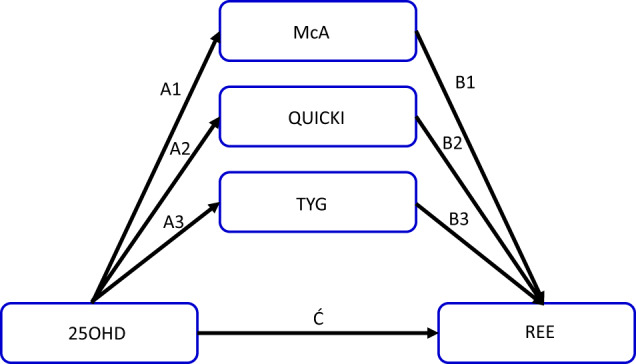
Model of mediation pathways of insulin sensitivity or insulin resistance on the association between vitamin D status and resting energy expenditure. A1, A2, A3 are the indirect pathways from 25OHD to each IS/IR index, while the corresponding paths B1, B2 B3 links each IS/IR to REE. Ć is the direct pathway from 25OHD to REE. Total effect of a model is given by C = [Ć + (A x B)]. 25OHD, 25dihydroxycholecalciferol; McA McAuleys index; QUICKI Quantitative insulin insensitivity check index; TYG Triglyceride and glucose index; REE Resting energy expenditure.

Mediation analysis has been widely reported in the nutrition literature [18–23]. When applied to RCTs or longitudinal cohort studies [18, 20], mediation models provide causal inferences between the intervention or exposure, and the clinical outcomes studied. However, mediation analysis has also been applied to cross sectional data [21–23]. In such situations, its purpose is to drive the development of hypotheses that advance nutrition concepts, and thereby provide a template for validation through future RCTs or cohort studies [21]. In this extended dataset, we have tested the direct and indirect relationships between 25OHD, IS/IR and REE. As there are many surrogate measures of IS/IR in the nutrition literature, we have modeled three common surrogate indices of IS/IR (Fig. 1) to ascertain their utility. We explored the hypothesis that part of the relationship between vitamin D and REE, was mediated through IS/IR (Fig. 1). By extension, if the mediation effect was negative and sizeable, it would dampen, perhaps nullify, the overall effect of the vitamin on REE.

## Methods

We collated the data on 155 Caucasian adults who had participated in clinical trials conducted in our laboratory. REE was measured by canopy mode indirect calorimetry (*n* = 119; Deltatrac II, Datex Instrumentarium, Finland or *n* = 36 TrueOne, Parvo Medics USA), using a standardized protocol that emphasized a 10–12 h overnight fast, 24 h abstinence from heavy physical activity, and a mandatory 30 min rest in the supine position prior to measurement [16]. The TrueOne provides valid measurements of REE when compared to the Deltatrac II [24], with excellent CVs for accuracy and reliability [25]. All measurements were conducted between 22–25 °C in a temperature-controlled room. Minute to minute recordings of O_2_ consumption and CO_2_ production were made over 30 min. Weir’s equation was used to calculate REE from the average of the last 25 min of data collection [26], and respiratory quotient (RQ) was measured by dividing CO_2_ production by O_2_ consumption over the same period. None of the participants in this collation had a measured RQ < 0.7 or >1.0 [27]. Body composition was assessed using dual energy X-ray absorptiometry (DEXA, DPX-L (*n* = 28) or Prodigy Models (*n* = 127), Lunar Corporation, USA) and a validation study has demonstrated equivalence in their estimates [28]. Fasting blood clinical chemistry measurements were conducted by the accredited laboratory of the Department of Pathology, Royal Perth Hospital, Perth WA. Vitamin D status (25OHD) was determined using the chemiluminescence immunoassay method (*n* = 69 Liaison, DiaSorin or *n* = 89 Architect, Abbott). The majority of measurements were made in singlicate, but flagged values were repeat tested for confirmation. The number of metabolic syndrome components for each individual were determined from criteria established from the last consensus guidelines of Alberti et al. [29]. McAuley’s insulin sensitivity index [30], QUICKI [30], and the triglyceride and glucose index [31] were calculated using established formulae.

### Participant Selection & Ethical standards

All participants in this analysis identified as Australians of European origin; were aged between 20 and 70 years, with a body mass index (BMI) ≥ 18.5 kg/m^2^. On detailed medical history, they had reported weight stability (over the last 6 months); not suffering from any medical conditions involving the thyroid, liver, kidney, or heart; absence of pregnancy; absence of PCOS, no history of cigarette smoking within a year prior to the study; not suffering from any current illness or infection requiring antibiotics; no gastrointestinal problems or history of gastrointestinal surgeries; no history of blood disorders; no history of mitochondrial disease; not on any medications that influence mitochondrial function (insulin, HMG-CoA reductase inhibitors, thiazolidinediones’), not on anti-convulsants, parathyroid hormone (PTH) or its derivatives, calcitonin, HRT, corticosteroids, testosterone replacement therapy, vitamin D supplements or any special or commercial diet programs that may affect metabolism. The Human Research Ethics Committee of the institute had approved all the studies used in the collation of data (HREC HR 20/2005; HR 103/2012; HR 4493/2013; RDHS-13-15). Each participant had provided written, informed consent, prior to their enrollment in those studies that were performed in accordance with the ethical standards laid down in the 1964 Declaration of Helsinki and its later amendments.

#### Statistical analysis

The aim of this analysis was to assess whether the association between 25OHD and REE was partly mediated by commonly used IS/IR indices (i.e., McA, QUICKI, or TYG) (Fig. 1), each being tested separately. The required sample size was calculated on the recommendations of Fritz and MacKinnon (2007) [32]. Based on a power of 80%, at 5% significance level, the minimum number needed to detect a small-to-medium effect of 0.26 for Path A (25OHD → possible mediator)(Fig. 1), and a medium effect of 0.39 for Path B (possible mediator → REE), was 126. We used *n* = 155 that was approximately 20% more than required. Data were analysed using IBM SPSS Statistics for Windows, version 25 (IBM Corp., USA) and mediation analysis used PROCESS SPSS Macro version 4.0 by Hayes [21] with a simple mediation model (Model No.4) to assess the mediating effects. The Bootstrapping method with 5000 samples was used to generate the 95% confidence interval for the mediating effects. An indirect mediation effect was defined as significant at the 5% level, if the 95% Bootstrap confidence interval did not encompass zero.

As a first step, we ran all models without any adjustment of covariates. The potential covariates available in our dataset included, age, gender, fat mass, fat free mass, season of measurement (winter/spring vs. summer/autumn), REE instrument used (Deltatrac vs. TrueOne) and vitamin D status method (Diasorin vs. Architect), and all individual components of MetS. Unmeasured variables with potential effects on REE and IS/IR, included a variety of hormones (adiponectin, cortisol, leptin, thyroxine, etc.) and unmeasured variables that determine 25OHD included sun exposure (min/d), sunscreen use and dietary/supplemental intake of vitamin D. In order to avoid selection and other biases, we constructed a directed acyclical graph (DAG), which helped determine the minimal set of covariates for the total effect. The DAGitty software (http://www.dagitty.net/) was used to construct the DAG [33]. We initially produced a generic DAG based on all measured variables that could be potentially important to exposure, mediator, and the outcome variable, REE. This DAG was then simplified to indicate the minimal set of covariates that were used in our second step to perform the adjusted mediation analysis. At the third step, we extended the minimal set of covariates by adding REE instrument usage and vitamin D testing method to the mediation analysis.

## Results

The general characteristics and metabolic features of the 96 women and 59 men are presented in Table 1. Following recruitment 75 participants were classified as metabolically normal (i.e., without MetS) and *n* = 80 were classified with MetS. Measurements of 25OHD revealed that *n* = 49 were inadequate in 25OHD with values <50 nmol/l, *n* = 67 were between 50–75 nmol/l and *n* = 39 had values >75 nmol/l. An overview of the hypothesis for direct and indirect mediation analysis using each IS/IR marker is presented in Fig. 1.

**Table 1:** General characteristics of the study population.

	Mean (SD)	Range
Age (yr)	50 (14.5)	19–80
Gender	59 M/ 96 F	–
FM (kg)	33.8 (11.23)	11.3–71.5
FFM (kg)	54.1 (12.32)	31.8–95.8
REE (kJ/d)	6487 (1322)	3422–10571
25OHD (nmol/l)	62.5 (20.8)	17–125.9
McAuley’s index (McA)	7.42 (2.070)	3.59–13.21
QUICKI	0.35 (0.036)	0.28–0.47
TYG	8.75 (.621)	7.4–10.4
TMetS	2.6 (1.29)	0–5
WC (cm)	100.3 (14.2)	71–146.7
Trig (mg/dl)	145 (89.5)	35.4–539.8
HDL (mmol/L)	1.4 (0.367)	0.7–2.4
SBP (mmHg)	127 (15.1)	93–183
DBP (mmHg)	75 (9.6)	47–96

*n* = 155; *SE* Standard error; *M* Male; *F* Female; *FM* Fat mass; *FFM* Fat free mass; *REE* Resting energy expenditure; *McA* McAuley’s index of insulin sensitivity; *QUICKI* Quantitative insulin sensitivity check index; *TYG* Triglyceride and glucose ratio; *TMetS* Total number of metabolic syndrome components; *WC* Waist circumference; *Trig* Triglycerides; *HDL* High density lipoprotein; *SBP* Systolic blood pressure; *DBP* Diastolic blood pressure.

Unadjusted Pearson’s correlation analysis showed significant correlations between McA and QUICKI (*r* = 0.856, *p* < 0.001), McA and TGY (*r* = −0.843, *P* < 0.001) and QUICKI and TYG (*r* = −0.537, *P* < 0.001). MCA and QUICKI were significantly and positively related to 25OHD, and negatively to REE. (Supplementary Table S1). TYG was significantly and negatively related to 25OHD, but positively to REE.

Unadjusted mediation analysis for direct effects, indirect mediation by each IS/IR and total effects are provided in Table 2. We noted the following:

1. The direct effect (Ć) of 25OHD on REE (Fig. 1) was small and non-significant when each surrogate IS/IR was modeled in turn.
2. There was a significant negative mediating effect with each IS/IR modeled.
3. The total effect (C) of all three models was negative and marginal in significance (*P* < 0.07).

**Table 2:** Unadjusted models of the mediating effects of surrogate measures of insulin sensitivity on the association between 25OHD and REE.

Hypothesized mediator	Effect of 25OHD on hypothesized mediator		Effect of hypothesized mediator on REE		Mediating effect of hypothesized mediator on association between 25OHD and REE		Direct effect of 25OHD on REE		Total effect of 25OHD on REE	
	A (SE)	*p*	B (SE)	*p*	AB (BootSE)	Bootstrap 95% CI	Ć (SE)	*p*	C (SE)	*p*
McA	0.037 (0.007)	<0.001	−310.3 (49.13)	<0.001	**−11.530 (3.081)**	**(−17.919, −5.924)**	2.13 (4.898)	0.664	−9.396 (5.090)	0.067
QUICKI	0.0005 (0.0001)	<0.001	−16919 .1 (2815.14)	<0.001	**−9.160 (2.677)**	**(−14.647, −4.139)**	−0.236 (4.837)	0.961	−9.396 (5.090)	0.067
TYG	−0.010 (0.002)	<0.001	731.3 (171.57)	<0.001	**−7.386 (2.197)**	**(−12.032, −3.491)**	−2.010 (5.128)	0.696	−9.396 (5.090)	0.067

*N* = 155; Values in bold are *p* < 0.05.

*25OHD* 25dihydroxycholecalciferol; *McA* McAuleys index; *QUICKI* Quantitative insulin insensitivity check index; *TYG* Triglyceride to glucose ratio. *SE* Standard error; *CI* Confidence interval; BootSE standard error obtained based on 5000 Bootstrap samples; Bootstrap 95% CI: 95% confidence interval generated based on 5000 Bootstrap samples.

**Fig. 2:**
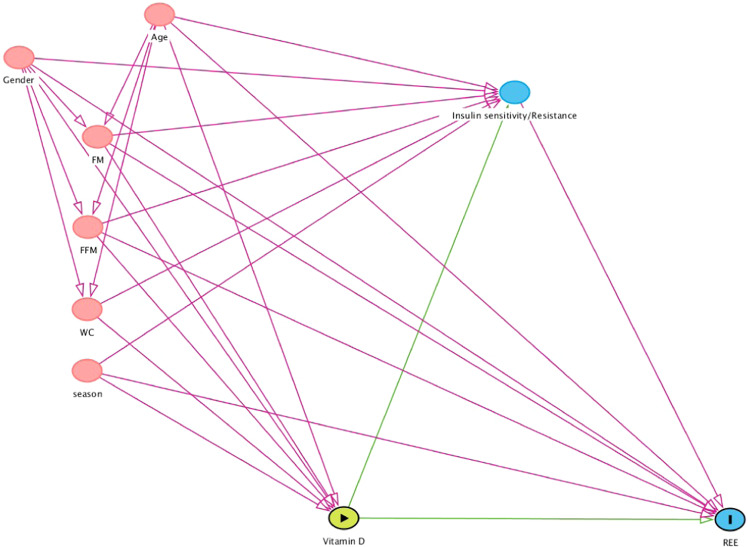
Simplified directed acyclical graph (DAG) of covariates influencing the causal pathway of vitamin D to REE. The simplified DAG included covariates age, gender, FM fatmass, FFM fat free mass, WC waist circumference, and season. Green line shows causal path and pink line biasing path.

The generic DAG, including all available variables potentially associated with 25OHD, IS/IR and REE, is illustrated in Supplementary Fig. S1. From a pathophysiological perspective, components of MetS are outcome variables; downstream of IS/IR. Depending on the MetS variables used to calculate IS/IR in this study (glucose, triglycerides, or both), the corresponding MetS component would need to be deleted from this figure to arrive at individual figures for each IS/IR. Clearly, this DAG (Supplementary Fig. S1) indicated that only age, gender, season, WC, FM, and FFM were the minimally required covariates for analysing the total effect. Accordingly, we deleted all unrequired and unmeasured variables, and reproduced a simplified DAG using the above minimal set of covariates (Fig. 2), that underscored the final adjusted analysis.

Table 3 provides the final adjusted results for direct effects, indirect mediation by each IS/IR and total effects. We noted the following:

1. A positive direct effect of 25OHD on REE that reached significance for models that included McA and TYG, but not QUICKI. The effect of 25OHD was represented by *β* coefficient: (SE) 4.8 (2.24), *p* = 0.035 for McA; 3.5 (2.24), *p* = 0.124 for QUICKI; 4.6 (2.26), *p* = 0.043 for TYG.
2. A significant negative mediating effect of McA on REE [β coefficient −2.1 (0.82) 95%CI −3.9,−0.70], and a significant negative mediating effect of TYG on REE of 63% [−1.93 (0.78) 95%CI −3.68,−0.62]. The mediation through QUICKI was also negative but its value was smaller relative to the other two and did not reach statistical significance (Table 3).
3. Accordingly, the total effect of the model (direct plus indirect) was positive but not statistically significant with each IS/IR tested (Table 3).

**Table 3:** Adjusted models^*^ for mediating effects of surrogate measures of insulin sensitivity on the association between 25OHD and REE.

Hypothesized mediator	Effect of 25OHD on hypothesized mediator		Effect of hypothesized mediator on REE		Mediating effect of hypothesized mediator on the association between 25OHD and REE		Direct effect of 25OHD on REE		Total effect of 25OHD on REE	
	A (SE)	*p*	B (SE)	*p*	AB (BootSE)	Bootstrap 95% CI	Ć (SE)	*p*	C (SE)	*p*
McA	0.024 (0.006)	0.0002	−88.250 (28.084)	0.002	**−2.100 (0.821)**	**(−3.934, −0.703)**	4.779 (2.239)	0.035	2.679 (2.201)	0.225
QUICKI	0.0003 (0.0001)	0.012	−2583.457 (1521.544)	0.092	−0.783 (0.534)	(−1.939, 0.134)	3.462 (2.235)	0.124	2.679 (2.201)	0.225
TYG	−0.007 (0.002)	0.0002	259.859 (91.764)	0.005	**−1.935 (0.780)**	**(−3.679, −0.622)**	4.614 (2.256)	0.043	2.679 (2.201)	0.225

*N* = 155. Values in bold are *p* < 0.05

*All models were adjusted for age, gender, FM, FFM, season, and waist circumference.

*25OHD* 25dihydroxycholecalciferol; *McA* McAuleys index; *QUICKI* Quantitative insulin insensitivity check index; *TYG* Triglyceride to glucose ratio.

*SE* Standard error; *CI* Confidence interval; BootSE standard error obtained based on 5000 Bootstrap samples; Bootstrap 95% CI: 95% confidence interval generated based on 5000 Bootstrap samples.

The additional analysis using the minimum set of covariates but adding both REE instrument and vitamin D testing method, is represented by Supplementary Table S2. The overall results of that analysis were similar to those in Table 3. A significant mediation effect was observed with McA and TYG but not QUICKI, and direct effects of 25OHD on REE were positive and closer to statistical significance (Supplementary Table S2).

## Discussion

The potential role of vitamin D in the regulation of body weight in humans has been reviewed [8, 34], with animal and cellular models suggesting a role for the vitamin in energy balance. However, the precise mechanisms underpinning the influence of the vitamin on human energy expenditure are yet to be confirmed. The present analysis was based on the hypothesis that insulin sensitivity (IS)/insulin resistance (IR) may mediate the association of vitamin D status (25OHD) on resting energy expenditure (REE). We observed a partial negative mediation of IS/IR on the positive relationship between higher vitamin D status and increased REE. The magnitude of this mediation indicated that any projected increases in REE following vitamin D supplementation, would be significantly dampened by concomitant improvements in IS or reductions in IR (Table 2).

1,25-dihydroxyvitamin D (1,25(OH)_2_D) is the major active form of the vitamin, and exerts its effects via the vitamin D receptor (VDR). Global VDR null mice, when compared to wild type mice, displayed a lower body weight even on a high fat diet [6]. This arose from a greater energy expenditure and an upregulation of uncoupling proteins (UCP) in adipose tissue. Another pathway for increased energy expenditure in VDR null mice could involve the higher bile acid pool seen in these animals [34]. Interestingly, targeted over expression of VDR in adipose tissue of mice, led to a higher body weight and specifically greater fat mass [7]. There was no change in food intake in these animals, but energy expenditure was reduced, partly from a suppression of UCP [7]. Further, cellular models of research indicate that treatment with 1,25(OH)_2_D suppressed UCP expression in primary cultures of brown adipose tissue (BAT) [6, 7]. Extrapolating outcomes from animal models to humans is not straightforward, and the discrepancies are not easily understood [34]. Currently, human studies that have addressed this area are few in number, and present their own issues. For example, a cross sectional study from Iran reported that 25OHD was positively related to REE/kg body weight [22]. Besides the manner of expression of REE, those authors further adjusted REE/kg weight for fat free mass and other covariates, which makes interpretation difficult. Another cross sectional study from the same country, where ~80% of the participants had 25OHD < 30 nmol/L, reported no relationship between 25OHD and REE adjusted for several covariates including a measure of IR [35]. There are three supplementation studies to date in the literature. A 1-week intervention found no effect on energy metabolism or substrate utilization, following vitamin D supplementation [36]. A longer 6-month trial was conducted on individuals with vitamin D deficiency and type 2 diabetes [37], where the supplementation followed a bolus dose regimen. The vitamin D supplemented group showed a small but significant decrease in absolute REE, but that data was not adjusted for any change in body composition over the trial period [37]. In addition, the authors reported that insulin sensitivity, as measured by the gold standard hyperinsulinemic-euglycemic clamp technique, was not different [37]. An acknowledged limitation of that study was a steady decrease in 25OHD over time. By trial completion at 6 months, the final status had a mean (SD) of 54 (9.2) nmol/l. There is a growing view that a threshold level of 25OHD > 75 nmol/L, is required for non-skeletal effects of the vitamin [11, 38, 39]. Vitamin D replete individuals (baseline 94 nmol/L) assigned to a treatment group, increased their 25OHD by ~30 nmol/L but showed no difference in their raised REE compared to the placebo group (baseline ~74 nmol/L) who showed a decline in their status. [40]. Indirect evidence in humans comes from the close relationship of mitochondrial oxygen affinity with REE [41], and that mitochondrial oxidative function of skeletal muscle improved with vitamin D supplementation [42]. Overall, consistent direct human evidence in support or against the hypothesis is lacking, and so future research is required to test the paradigm generated here.

Previous RCTS investigating the impact of vitamin D on IS/IR had been critiqued for their methodological flaws and other limitations. Mostly, these RCTS were not primarily designed for evaluating glycaemic-related outcomes, and they used inappropriate or infrequent doses that did not sustain vitamin D concentration. A recent meta-analysis that addressed these concerns was based on 28 RCTs identified through a systematic review. The author showed a reduction in IR following vitamin D supplementation [43]. There are several potential mechanisms, that may underscore this outcome, and its subsequent impact on energy metabolism. Vitamin D may increase insulin secretion via modulation of calcium concentration in pancreatic *β* cells [17, 44]. Vitamin D could also directly decrease pro-inflammatory cytokines, which are associated with IR [45]. Cytokines act through plasma cell membrane receptors to increase energy expenditure [46], so any decrease in pro-inflammatory cytokines, would reduce energy expenditure. The older literature in support comes from observations that basal endogenous glucose output, fasting insulin, free fatty acid concentrations, and glucose disposal were all significant determinants of REE [47]. An observational human study also found a positive relationship between changes in fasting glycemia, and changes in REE, 24 h energy expenditure and sleeping metabolic rate, respectively; where greater hyperglycemia promoted higher rates of energy expenditure and a reduced risk of weight gain [48].

In this mediation analysis, we found consistent evidence for a sizeable direct pathway between 25OHD and REE, however, only 2 of 3 adjusted models showed a statistically significant effect (Table 3). There was also a significant, negative mediation pathway through both McA and TGY, but not with QUICKI. Those negative mediation effects blunted the overall effect across all markers of IS/IR (Table 3). The absence of a significant mediation effect with QUICKI was unexpected. All three IS/IR variables were significantly related to each other, and to REE and 25OHD, respectively, in a simple correlation analysis (Supplementary Table S1). Clearly the mediation effect, as a proportion of the direct effect, was ~50% lower for QUICKI in the fully adjusted model, in comparison to the other two surrogate measures (Table 3). So, we lacked the power to detect this unexpectedly smaller effect. However, it was also likely that all surrogate markers of IS/IR were not equally efficient in predicting changes in energy expenditure. Other authors working on different clinical endpoints have reached similar conclusions [49–51]. They too noted significant simple correlations between all markers and their study endpoints (e.g., metabolic factors [49], cardiovascular disease [50], or type 2 diabetes [51]), but with fully adjusted models the predictive ability of each surrogate marker used in their studies, was quite different. The way forward in this area would be the inclusion of several surrogate markers, especially when studying other global populations/ethnic groups.

## Limitations

These cross sectional data, restricted to one ethnic group, are not representative of the present Australian population nor of other populations groups worldwide. Some unexpectedly small effect sizes were not detected, with resultant *p* values <10%. While this is acceptable for hypothesis generation, a much larger cohort would have overcome this limitation. Moreover, a larger sample would have allowed the modeling to be developed on one random half, and validated on the other half within this paper. We have identified some unmeasured variables, mainly circulating hormones like thyroid, parathyroid hormone, leptin, cortisol, adiponectin, etc. that could make small contributions to residual variation in REE, and impinge on IS/IR as well. Their inclusion in future studies could be important to clarifying the precise contributions of 25OHD and IS, to REE.

## Conclusions

We hypothesize a sizeable, partial negative mediation of IS/IR on the close direct relationship of vitamin D status to REE. Projected increases in REE following vitamin D supplementation, are hence likely to be significantly dampened by concomitant improvements in IS, or reductions in IR. When assessing the role of vitamin D on energy metabolism, and perhaps on other outcomes of chronic disease, our suggestion is to test several measures of IS/IR in the analysis. Future vitamin D supplementation trials with adequate sample sizes, and addressing the limitations highlighted here could validate the causal framework presented.

## Supplementary information

Legend for supplementary Tables & Figures

Figure S1

Table S1

Table S2

## Data Availability

The raw data used in this paper is freely available to any researcher wishing to use them for non-commercial purposes, provided the institution’s human ethics committee approves the request.
