# Supplementary material for: Hypothesized pathways for the association of vitamin D status and insulin sensitivity with resting energy expenditure: a cross sectional mediation analysis in Australian adults of European ancestry
Source: Eur J Clin Nutr. 2022 Apr 1;76(10):1457–63. doi: 10.1038/s41430-022-01123-4 (PMC9550620; doi:10.1038/s41430-022-01123-4)
Supplement: Supplementary file 1 — Legend for supplementary Tables & Figures [file 41430_2022_1123_MOESM1_ESM.docx]

**Title & Legends for Supplementary Tables & Figure:**

Table S1: Pearson’s correlation coefficients between outcome, mediator variables and REE

Legend: REE, resting energy expenditure; 25OHD, 25dihydroxycholecalciferol; McA, McAuleys index; QUICKI, quantitative insulin sensitivity check index; TYG, triglyceride and glucose index.

Table S2: Additional adjustment^*^ for mediating effects of insulin sensitivity on the association between 25OHD and REE.

**Legend:**

**^*^**All models were adjusted for age, gender, FM, FFM, season, waist circumference, and additionally for methods of REE as well as 25OHD measurement. N=155. Values in bold are p <0.05

SE: standard error; CI: confidence interval; BootSE**:** standard error obtained based on 5000 Bootstrap samples; Bootstrap 95% CI: 95% confidence interval generated based on 5000 Bootstrap samples.

25OHD, 25dihydroxycholecalciferol; McA, McAuleys index; QUICKI, quantitative insulin sensitivity check index; TYG, triglyceride to glucose ratio.

Figure S1: Composite directed acyclical graph (DAG) of all potential variables influencing the causal pathway of vitamin D to REE.

Legend: The composite DAG included measured covariates: age; gender; *FM,* fat mass; *FFM,* fat free mass; season; unmeasured covariates: hormones and sun exposure/diet; exposure antecedent: method of 25OHD; outcome antecedents: method of REE; other outcomes of IS/IR: *FBG,* fasting blood glucose; *TAG,* triacylglycerol; *HDL-C,* high density lipoprotein; *SBP,* systolic blood pressure*; DBP,* diastolic blood pressure.

green line =causal path; pink line =biasing path
