# Supplementary figures and images for: Hypothesized pathways for the association of vitamin D status and insulin sensitivity with resting energy expenditure: a cross sectional mediation analysis in Australian adults of European ancestry
Source: Eur J Clin Nutr. 2022 Apr 1;76(10):1457–63. doi: 10.1038/s41430-022-01123-4 (PMC9550620; doi:10.1038/s41430-022-01123-4)

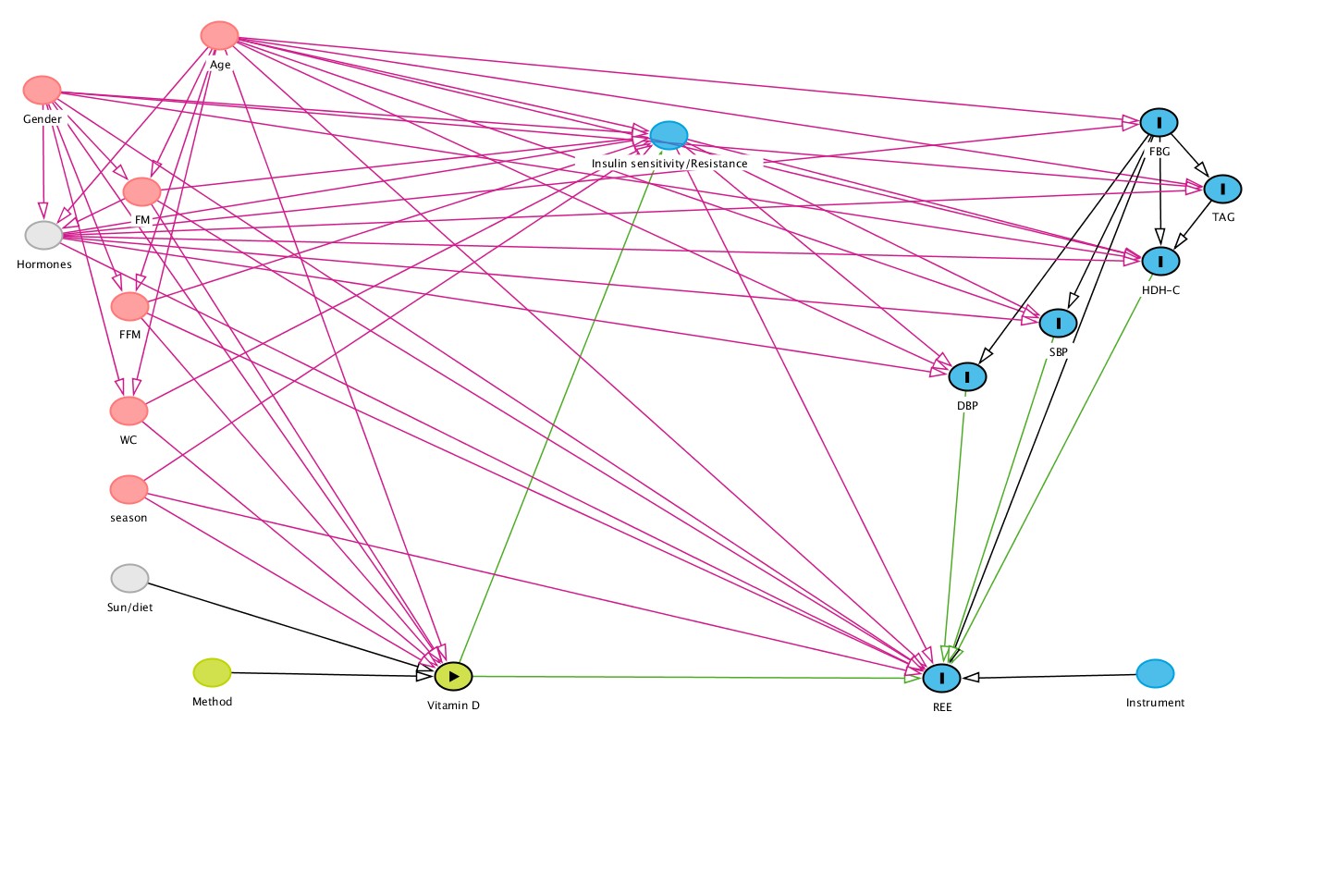

Supplement: Supplementary file 2 — Figure S1 [file 41430_2022_1123_MOESM2_ESM.jpg]
