# Supplementary material for: Hypothesized pathways for the association of vitamin D status and insulin sensitivity with resting energy expenditure: a cross sectional mediation analysis in Australian adults of European ancestry
Source: Eur J Clin Nutr. 2022 Apr 1;76(10):1457–63. doi: 10.1038/s41430-022-01123-4 (PMC9550620; doi:10.1038/s41430-022-01123-4)
Supplement: Supplementary file 3 — Table S1 [file 41430_2022_1123_MOESM3_ESM.docx]

Supplementary Table S1: Pearson’s correlation coefficients between outcome, mediator variables and REE

| Variable | Mean ± SD | Pearson’s Correlation Coefficient r (p value) | | | | |
| --- | --- | --- | --- | --- | --- | --- |
|  |  | REE | 25OHD | McA | QUICKI | TYG |
| REE | 6486.56 ± 1321.10 | 1 | - | - | - | - |
| 25OHD | 62.53 ± 20.77 | -0.148 (0.067) | 1 | - | - | - |
| McA | 7.42 ± 2.07 | **-0.473 (<0.001)** | **0.373 (<0.001)** | 1 | - | - |
| QUICKI | 0.35 (0.003) | **-0.458 (<0.001)** | **0.315 (<0.001)** | **0.856 (<0.001)** | 1 | - |
| TYG | 8.75 (0.05) | **0.354 (<0.001)** | **-0.338 (<0.001)** | **- 0.843 (<0.001)** | **-0.537 (<0.001)** | 1 |

Legend: REE, resting energy expenditure; 25OHD, 25dihydroxycholecalciferol; McA, McAuleys index; QUICKI, quantitative insulin sensitivity check index; TYG, triglyceride and glucose index.
