# Supplementary material for: Hypothesized pathways for the association of vitamin D status and insulin sensitivity with resting energy expenditure: a cross sectional mediation analysis in Australian adults of European ancestry
Source: Eur J Clin Nutr. 2022 Apr 1;76(10):1457–63. doi: 10.1038/s41430-022-01123-4 (PMC9550620; doi:10.1038/s41430-022-01123-4)
Supplement: Supplementary file 4 — Table S2 [file 41430_2022_1123_MOESM4_ESM.docx]

Suppl Table S2. Additional adjustment^*^ for mediating effects of insulin sensitivity on the association between 25OHD and REE.

| Hypothesized mediator | Effect of 25OHD on hypothesized mediator | | Effect of hypothesized mediator on REE | | Mediating effect of hypothesized mediator on the association between 25OHD and REE | | Direct effect of 25OHD on REE | | Total effect of 25OHD on REE | |
| --- | --- | --- | --- | --- | --- | --- | --- | --- | --- | --- |
|  | A (SE) | p | B (SE) | p | AB (BootSE) | Bootstrap 95% CI | Ć (SE) | p | C (SE) | p |
| McA | 0.0225 (0.0064) | 0.0005 | -93.44 (28.35) | 0.0012 | **-2.099 (0.808)** | **(-3.845, -0.675)** | 4.05 (2.26) | 0.075 | 1.95 (2.24) | 0.385 |
| QUICKI | 0.0003 (0.0001) | 0.025 | -2890 (1548.42) | 0.064 | -0.778 (0.527) | (-1.947, 0.0997) | 2.73 (2.26) | 0.229 | 1.95 (2.24) | 0.385 |
| TYG | -0.0068 (0.002) | 0.0006 | 283.68 (93.50) | 0.0026 | **-1.936 (0.765)** | **(-3.639, -0.645)** | 3.88 (2.269) | 0.088 | 1.95 (2.24) | 0.385 |

**Legend:**

**^*^**All models were adjusted for age, gender, FM, FFM, season, waist circumference, and additionally for methods of REE as well as 25OHD measurement. N=155. Values in bold are p <0.05

SE: standard error; CI: confidence interval; BootSE**:** standard error obtained based on 5000 Bootstrap samples; Bootstrap 95% CI: 95% confidence interval generated based on 5000 Bootstrap samples.

25OHD, 25dihydroxycholecalciferol; McA, McAuleys index; QUICKI, quantitative insulin sensitivity check index; TYG, triglyceride to glucose ratio.
